# Exploring the molecular mechanism of *OsROS1a* in regulating resistance to bacterial leaf streak through transcriptome and DNA methylation profiling in rice (*Oryza sativa* L.)

**DOI:** 10.1186/s12864-025-11895-1

**Published:** 2025-08-01

**Authors:** Xiaofang Xie, Xuansong Yang, Libin Lu, Tong Li, Mingyue Qin, Huazhong Guan, Yan Zheng, Tao Lan, Weiren Wu

**Affiliations:** 1 College of Life Sciences, Fujian Agriculture & Forestry University, Fuzhou, 350002 China; 2 Fujian Key Laboratory of Crop Breeding by Design, Fujian Agriculture & Forestry University, Fuzhou, 350002 China; 3 Fujian Academy of Agricultural Sciences, Fuzhou, 350003 China

**Keywords:** *OsROS1a*, Rice, Bacterial leaf streak, Transcriptome, Methylation

## Abstract

**Background:**

DNA demethylases regulate the levels of genomic DNA methylation in plants. The demethylase REPRESSOR OF SILENCING 1 (ROS1) is a crucial factor for modulating gene expression in plant disease responses. Bacterial leaf streak (BLS), caused by *Xanthomonas oryzae* pv. Oryzicola (*Xoc*), is a highly destructive disease in rice. BLS resistance in rice is known to be quantitatively inherited, but the mechanisms by which DNA methylation controls BLS resistance remain poorly understood.

**Results:**

In this study, we knocked down *OsROS1a* expression in the rice variety Nipponbare using RNA interference (RNAi). The average BLS lesion length in the transgenic (T2) *OsROS1a*-RNAi (RS) lines was significantly reduced compared to that in wild-type Nipponbare plants (NP). Using whole-genome bisulfite sequencing (WGBS) and RNA-sequencing (RNA-seq), we analyzed the DNA methylations and transcriptomes of RS lines in comparison with NP at 0 (control), 5, 10, and 24 h post-inoculation with *Xoc*. A total of 1080 differentially expressed genes (DEGs) related to *Xoc* infection between the NP and RS lines were identified, which could be grouped into 8 clusters by K-means analysis. The DEGs in cluster 1 were enriched in the biological process related to defense response, response to stress, oxidation-reduction, etc. Integration of the methylome and transcriptome data revealed 112 overlapping differentially methylated and expressed genes (DMEGs). Gene Ontology (GO) analysis showed that the DMEGs were mainly involved in biological processes, such as metabolic process, cellular process, responses to stimulus, signaling, and immune system processes. KEGG pathway enrichment analysis revealed that these DMEGs were enriched in pathways related to glutathione metabolism, plant-pathogen interaction, cysteine and methionine, diterpenoid biosynthesis, photosynthesis, and starch and sucrose. Additionally, *LOC_Os09g12660*, encoding the glucose-1-phosphate adenylyl transferase large subunit, a chloroplast precursor involved in synthesizing activated glycosyl donor, showed strong potential to contribute to BLS resistance.

**Conclusion:**

*OsROS1a* plays a crucial role in modulating rice resistance to bacterial leaf streak (BLS) caused by *Xanthomonas oryzae* pv. oryzicola (*Xoc*). These findings provide valuable insights into the role of *OsROS1a* in BLS resistance.

**Supplementary Information:**

The online version contains supplementary material available at 10.1186/s12864-025-11895-1.

## Background

DNA methylation is an epigenetic modification that occurs throughout the genome and plays a crucial role in regulating gene expression related to plant growth, development, and defense mechanisms [1–5]. In plants, DNA methylation occurs at CG, CHG, and CHH sequences (where H represents A, C, or T) [6, 7]. Cytosine methylation is reversible through demethylation processes [8, 9]. DNA demethylation can be classified into two modes: passive and active. Active demethylation is independent of the DNA replication cycle and involves the enzymatic removal of methylated cytosine [10, 11]. The REPRESSOR OF SILENCING 1 (ROS1)/DEMETER (DME; DEMETER-like, DML) family of DNA demethylases, known as glycosylases, plays a key role in DNA demethylation in plants [7]. They act as suppressors of transcriptional gene silencing by catalyzing the removal of 5-mC. *ROS1* encodes a bifunctional DNA demethylase that removes 5-methylcytosine and introduces nicks in double-stranded DNA [12]. *ROS1* is also known to negatively regulate the RNA-directed DNA methylation (RdDM) pathway [13, 14]. ROS1-mediated DNA demethylation is crucial for establishing genomic DNA methylation patterns and protects active genes from being silenced [15]. In *Arabidopsis*, the *ros1* mutant shows hypersensitive to ABA, and *ROS1* participates in the ABA response by regulating the expression of *NICOTINAMIDASE 3* (*NIC3*) [16]. It has been confirmed that *ros1* does not exhibit any obvious morphological changes and affect the global methylation status except for increasing methylation at specific loci [17]. The rice (*Oryza sativa* L.) genome contains four *ROS1* orthologs: *OsROS1a*, *b*, *c*, and *d* [18]. Among these genes, *OsROS1a* is indispensable for the development of gametophytes [19].

DNA methylation plays a crucial role in the regulation of gene expression and significantly contributes to plants’ ability to respond to stress through demethylation or *de novo* methylation [20]. For instance, DNA methylation level decreases under chilling stress, with differentially methylated genes involved in regulating transcription factor families [21]. In *Brassica rapa*, cold acclimation modifies DNA methylation patterns, which enhances tolerance to heat and improves growth rate [22]. Similarly, DNA methylation under freezing stress regulates various metabolic pathways related to cold tolerance in rapeseed [23]. In tea plants, significant loss of DNA methylation occurs at promoter and gene body regions after chilling stress [24].

For biotic stress, increasing evidence suggests that epigenetic processes involving methylation and demethylation play crucial roles in the regulation of gene expression in the plant response to disease [13, 25]. Recent studies have unveiled a correlation between pathogen-induced DNA hypomethylation and the transcriptional activation of nearby genes, implying a role of DNA methylation in modulating pathogen-induced gene expression [26]. Moreover, studies in *Arabidopsis* have indicated that DNA methylation is involved in the plant-induced immune response, and plays a role in activating the transcription of specific defense-related genes [3, 13]. Interspecific grafting dynamically adjusts the balance between growth and defense resources in the scion by altering gene methylation levels, thereby enhancing disease resistance at the cost of growth inhibition [27]. Additionally, age-related increases in methylation have been associated with improved resistance against the blight pathogen *X. oryzae* in rice [28]. Research has also shown that *DME* plays a crucial role in defense against the fungal pathogen *Verticillium dahlia* [29] and the bacterial pathogen *Pseudomonas syringae* pv. *tomato* (Pst). Mutants of *DME* exhibits increased susceptibility to both bacterial and fungal pathogens [30].

Rice is an important crop, and its yield is severely affected by bacterial leaf streak (BLS), a destructive disease caused by *Xanthomonas oryzae* pv. *Oryzicola*. BLS resistance in rice is quantitatively inherited, controlled by multiple quantitative trait loci (QTLs) with minor effect [31, 32]. The precise details of the typical horizontal resistance mechanism of BLS remain largely unknown [33]. The current understanding of the genetic basis of plant horizontal resistance is primarily limited to the identification and characterization of QTLs. As an important way of gene expression regulation, DNA methylation may also play a role in BLS resistance. Hence, studying the effect of DNA methylation on BLS resistance will promote our understanding of the molecular mechanisms regulating BLS resistance in rice. According to the Rice Gene Expression database (http://rice.plantbiology.msu.edu/expression.shtml), *OsROS1a* is the most extensively expressed gene among the four *ROS1* homologs (*OsROS1a*, *b*, *c*, and *d*) in rice. So, we focused on this gene. As knockout of *OsROS1a* leads to sterility [19, 34, 35], which was also observed in our experiment, we created reproducible and inheritable plant materials for this study by knocking down *OsROS1a* expression using RNA interference (RNAi). In this study, we performed a comprehensive analysis of the effects of *OsROS1a*-RNAi on gene expression regulation and transcriptional response to BLS pathogen *Xoc*, using DNA methylation and transcriptome profiling. The objectives of this study were to examine whether and how *OsROS1a* affects BLS resistance in rice, and to investigate the regulatory role and the molecular mechanism of *OsROS1a*-mediated BLS resistance from the perspective of DNA methylation. Our findings will not only offer new insights into the genetic basis of rice BLS resistance but also provide a foundation for developing rice varieties with enhanced resistance to this disease.

## Materials and methods

### Vector construction and rice transformation

**Fig. 1:**
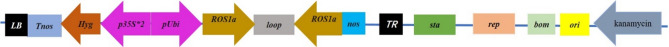
Schematic diagram of *OsROS1a*-RNAi construct. Detailed illustration of the *OsROS1a*-RNAi construct, including the key components and their arrangement

The *japonica* rice cultivar Nipponbare (NP), which is susceptible to BLS, was used in this study. To construct *OsROS1a*-RNAi (RS) vector, a 480-bp fragment containing part of the coding sequence of *LOC_Os01g11900* was amplified from the cDNA of Nipponbare by PCR, and was inserted into the pBWA(V)HU vector downstream of the ubiquitin promoter, in both forward and reverse orientations (Fig. 1). *Agrobacterium*-mediated transformation of Nipponbare was performed using the *OsROS1a*-pBWA(V)HU construct to obtain T_0_ plants [36]. Positive transgenic plants were screened by PCR using the hygromycin specific primers and quantitative real-time PCR (qRT-PCR) analysis. Fifteen positive T_2_ plants from each line were obtained for agronomic trait evaluation and BLS-resistance assessment. All the primers used for RS vector construction and transgenic line validation are shown in Additional file1 Table S1.

### Pathogen infection and resistance assessment

The *Xanthomonas oryzae* pv. oryzicola (*Xoc*) strain utilized to evaluate BLS resistance was kindly supplied by Prof. Guoying Cheng of Huazhong Agricultural University. Inoculation of rice leaves at the active tillering stage was conducted using the pricking method [37]. The lesion length on each plant was measured 24 days post-inoculation. The average lesion length of 15 plants (with three leaves scored per plant) was used to assess the resistance level of a particular line.

### Quantitative real-time PCR analysis

Total RNA extraction and first-strand cDNA synthesis were performed as described [32]. The relative expression level of genes was detected by qRT-PCR using SYBR Premix Ex Taq (Takara) and the 2^−ΔΔCt^ method [38] with *Actin* gene as the internal control. Each selected gene was assessed with three biological replicates and three technical replicates. The paired t-test method was used to analyze differences in gene expression levels between samples. The gene-specific primers employed for qRT-PCR analysis are listed in Additional file 2 Table S2.

### RNA sequencing and data analysis

Leaves of NP and one RS transgenic line (R-4) were inoculated with *Xoc*, and total RNA samples were collected from the leaves at 0 (as control), 5, 10, and 24 h post-inoculation, respectively. In total, there were eight treatments, denoted as NP0, NP5, NP10, NP24, RS0, RS5, RS10, and RS24, each with three biological replicates, resulting in 24 RNA samples. Each sample consisted of three 1.5-cm leaf segments adjacent to the inoculation site, collected from three individual plants. The RNA samples were sequenced using Illumina HiSeq 2500 by Biomarker Technologies (http://www.biomarker.com.cn/, BioMarker, Beijing, China), and the reads were mapped to the reference genome available in the Rice Genome Annotation Project database (http://rice.plantbiology.msu.edu). For gene expression analysis, the read counts were normalized using FPKM (Fragments Per Kilobase per Million mapped fragments) method [39]. Differentially expressed genes (DEGs) were identified based on the criteria of |log_2_(fold change)| ≥ 1 and false discovery rate (FDR) ≤ 0.01. DEGs related to *Xoc* infection between NP and RS were analyzed by K-means, gene ontology (GO) and KEGG analyses. The Gene Ontology Database (http://www.geneontology.org/) was used for GO analysis. GO terms with a corrected P-value < 0.05 were regarded as significant enrichment. The KEGG compound database (https://www.kegg.jp/kegg/compound/) was used to annotate the different metabolites, which were mapped to the KEGG pathway database (https://www.kegg.jp/kegg/pathway.html), enrichment with a P-value < 0.05 was considered significant. The MapMan tool (http://MapMan.gabipd.org) was used for a graphical overview of pathways involving the DEGs.

### Genomic bisulfite sequencing and data analysis

Leaves of NP and RS lines were inoculated with *Xoc*, and samples were collected at 0 (control), 5, 10, and 24 h post-inoculation. A total of eight DNA samples were used for cytosine methylome analysis. Genomic DNA extraction was performed using the Plant DNeasy kit (Qiagen) following the manufacturer’s protocol, and Bisulfite sequencing (BS-seq) libraries were prepared as described [40]. The bisulfite conversion efficiency was tested using the Lambda genome, with all samples showing a bisulfite conversion rate exceeding 99.5% [41]. The converted DNA fragments were subsequently amplified by PCR and sequenced using Illumina HiSeq TM 2500 by Gene Denovo Biotechnology Co. (Guangzhou, China). Following data filtering, the clean reads were mapped to the rice reference genome obtained from the Rice Genome Annotation Project database (http://rice.plantbiology.msu.edu) using BSMAP software (version: 2.90) [42]. Only the CG/CHG/CHH sites with a read coverage ≥ 5 across all samples were used for differential methylation assessment. Differentially methylated regions (DMRs) were identified based on the following criteria: regions with a q-value < 0.05, a minimum of five CG/CHG/CHH sites, and a methylation difference > 20%. The genome regions were classified as upstream 2 kb, 5′ untranslated region (UTR), 3′ UTR, genebody, downstream 2 kb, and transposon (TE). Violin plots were used to visually present the distribution of continuous variables across different categories, utilizing the R package ggplot2 (https://ggplot2.tidyverse.org). Additionally, the enrichment analyses of GO terms (http://systemsbiology.cau.edu.cn/agriGOv2/) and KEGG pathways (http://structuralbiology.cau.edu.cn/PlantGSEA/analysis.php) were also conducted for the differentially methylated genes (DMGs) and DEGs located within the DMRs (DEMGs), enrichment with a P-value < 0.05 was regarded as significant.

## Results

### Effect of repressing *OsROS1a* expression on resistance to *Xoc*

**Fig. 2:**
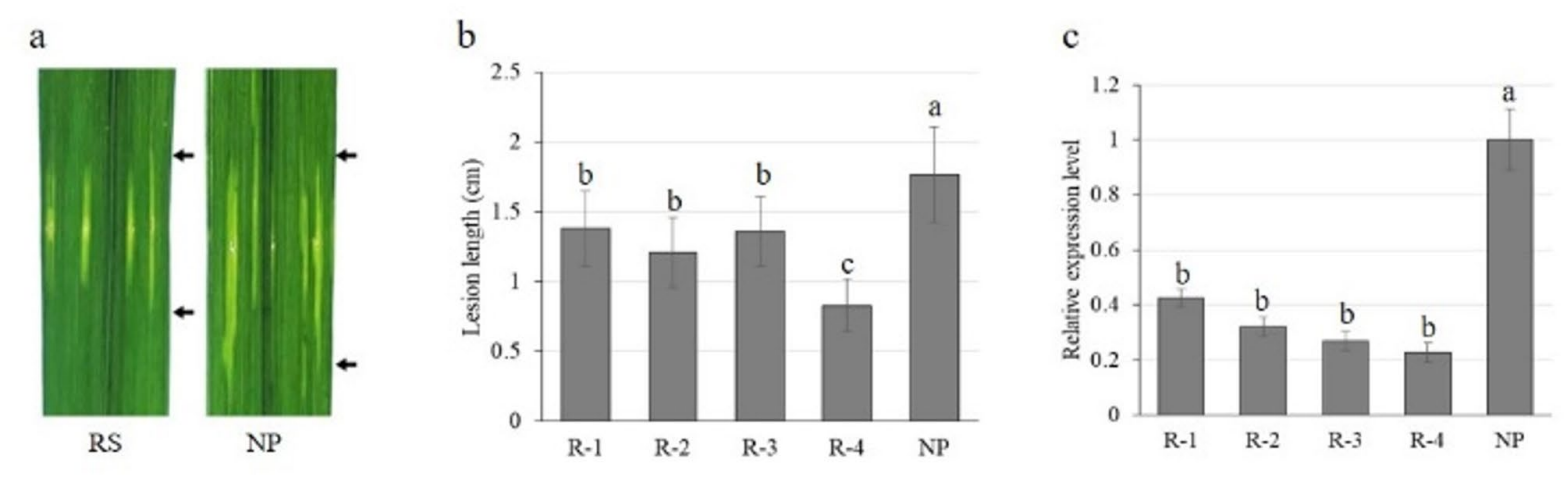
BLS resistance performance in *OsROS1a*-RNAi T_2_ lines and wild type Nipponbare (NP). **a** Leaves of WT (NP) and *OsROS1a*-RNAi line with BLS lesions at 24 d after *Xoc* inoculation. **b** Lesion lengths in NP and T_2_ *OsROS1a*-RNAi lines (RS-1/2/3/4) measured at 24 d after *Xoc* inoculation. **c** Relative expression levels of *OsROS1a* in leaves of NP and T_2_ *OsROS1a*-RNAi lines. Letters represent statistical significance of the difference at 0.05 level according to ANOVA-Tukey’s test

A total of 7 independent transgenic (T_0_) RS plants were obtained, from which four T₂ RS lines (R-1, R-2, R-3, and R-4) were subsequently selected. qRT-PCR analysis of positive T_2_ plants of each line showed that *OsROS1a* expression was significantly decreased in the RS lines (Fig. 2c). To investigate the relationship between *OsROS1a*-mediated DNA demethylation and BLS resistance, T_2_ RS lines were exposed to the bacterial pathogen *Xoc*. The result showed that the average lesion lengths in the RS lines were significantly reduced in comparison with that in WT (NP) plants (Fig. 2a and b). These results suggested that suppressing the expression of *OsROS1a* enhanced BLS resistance.

### Differentially expressed genes between the RS line and wild type related to *Xoc* infection

More than 40 million reads were obtained from each sample in the RNA-seq analysis on *Xoc*-inoculated RS and WT plants at 0, 5, 10, 24 h post-inoculation, and approximately 96% of the reads were successfully mapped to the Nipponbare reference genome, with more than 86% being uniquely mapped (Table 1). The RNA-seq data have been submitted to the database of the NCBI Sequence Read Archive (http://trace.ncbi.nlm.nih.gov/Traces/sra) under the accession number PRJNA1149540.

**Table 1:** Statistics of RNA-seq reads and alignment quality

Samples	Total reads	Mapped reads	Uniq mapped reads
NP0-1	68,637,672	66,429,481 (96.78%)	64,477,688 (93.94%)
NP0-2	57,912,782	55,600,607 (96.01%)	50,333,252 (86.91%)
NP0-3	64,069,316	62,001,769 (96.77%)	60,211,781 (93.98%)
NP10-1	60,216,530	58,341,459 (96.89%)	56,621,259 (94.03%)
NP5-1	55,761,664	54,108,610 (97.04%)	52,270,587 (93.74%)
NP5-2	59,233,238	57,371,532 (96.86%)	55,491,902 (93.68%)
NP5-3	54,333,532	52,502,550 (96.63%)	50,920,367 (93.72%)
NP10-2	48,502,796	47,013,871 (96.93%)	45,627,210 (94.07%)
NP10-3	57,875,530	55,867,351 (96.53%)	53,975,030 (93.26%)
NP24-1	53,412,648	51,562,100 (96.54%)	49,745,873 (93.14%)
NP24-2	50,353,466	48,822,292 (96.96%)	47,330,191 (94.00%)
NP24-3	50,905,806	49,227,271 (96.70%)	47,572,088 (93.45%)
SR0-1	52,898,166	50,541,522 (95.54%)	47,908,448 (90.57%)
SR0-2	54,650,920	52,607,136 (96.26%)	49,924,215 (91.35%)
SR0-3	50,401,206	48,536,688 (96.30%)	47,181,190 (93.61%)
SR5-1	55,809,580	53,815,037 (96.43%)	52,224,774 (93.58%)
SR5-2	64,071,834	62,080,195 (96.89%)	60,154,967 (93.89%)
SR5-3	75,137,178	72,441,884 (96.41%)	69,739,544 (92.82%)
SR10-2	53,409,816	51,489,613 (96.40%)	47,678,573 (89.27%)
SR10-1	65,392,436	63,463,822 (97.05%)	61,205,307 (93.60%)
SR10-3	42,325,600	40,991,285 (96.85%)	39,657,459 (93.70%)
SR24-1	43,286,422	41,977,576 (96.98%)	40,597,837 (93.79%)
SR24-2	56,311,182	54,625,227 (97.01%)	53,001,762 (94.12%)
SR24-3	54,447,246	52,827,524 (97.03%)	51,324,192 (94.26%)

**Fig. 3:**
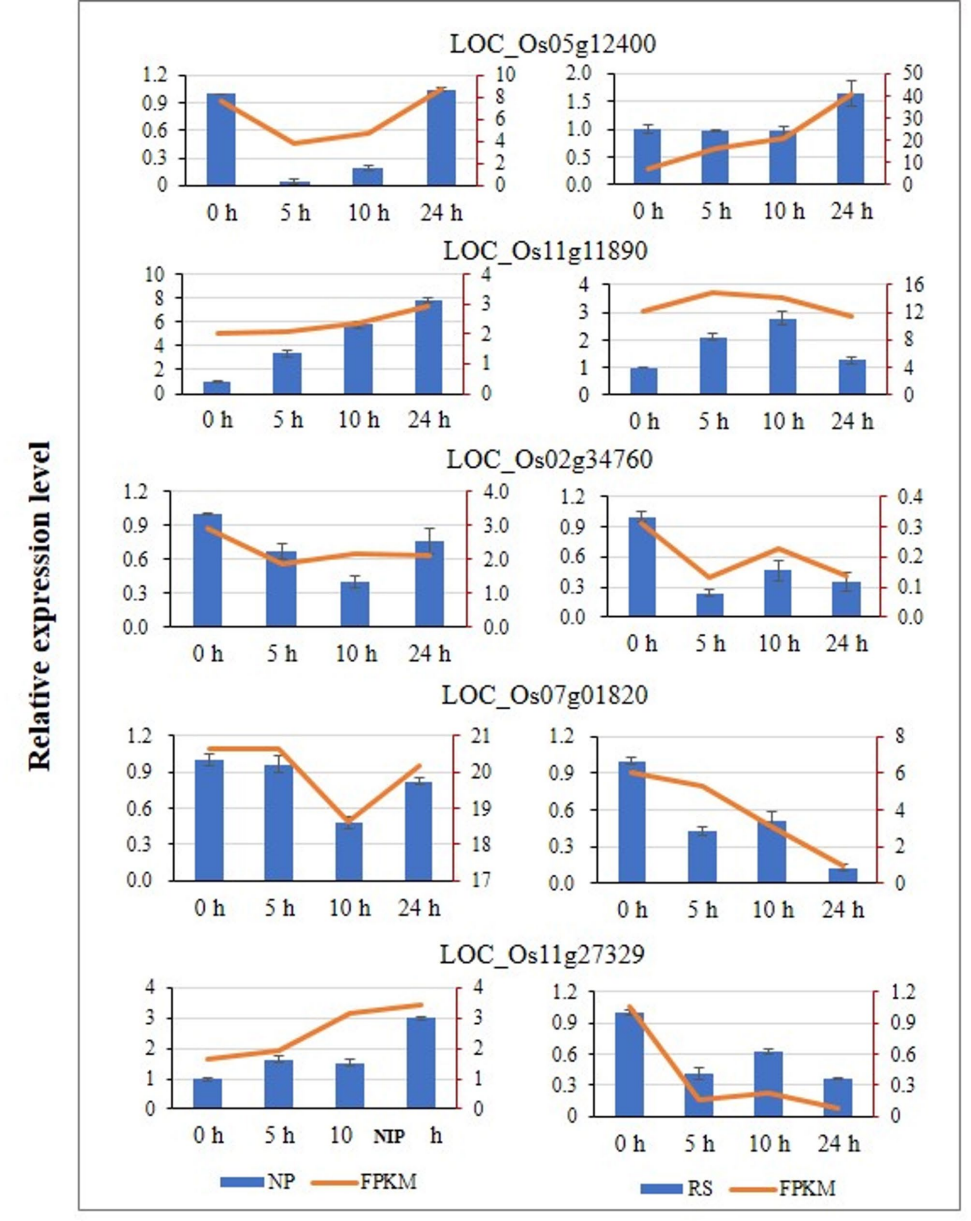
Quantitative RT-PCR analysis of five selected genes in leaves of wild type (NP) and *OsROS1a*-RNAi line (RS) to validate the RNA-seq data

qRT-PCR analysis of 5 genes encoding proteins related to disease resistance in the four treatments showed that their expression patterns detected by qRT-PCR were consistent with those detected by RNA-seq (Fig. 3). The results confirmed the reliability and suitability of the RNA-seq data for further transcriptome analysis.

**Fig. 4:**
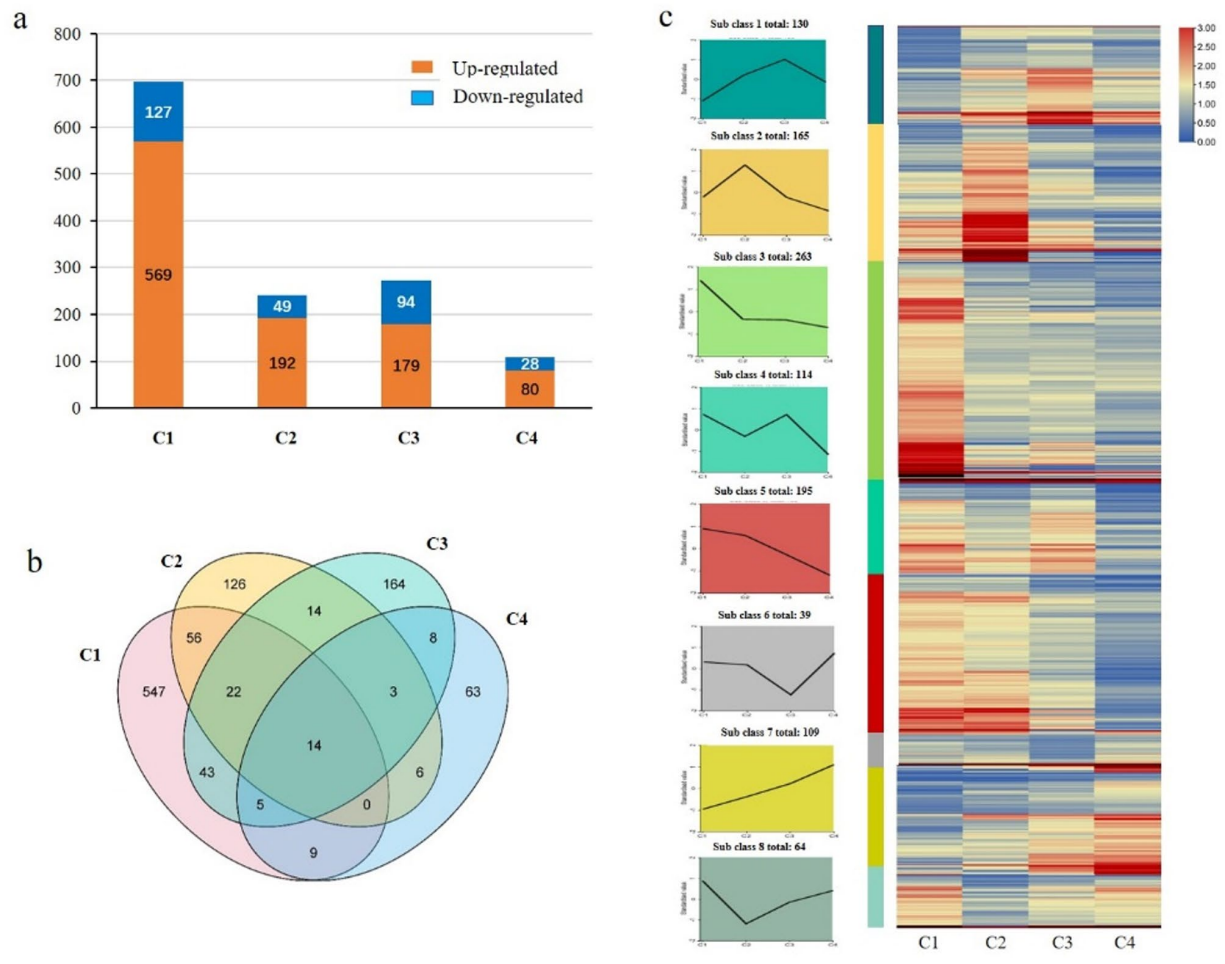
DEGs in each horizontal comparison group overlapping with the longitudinal comparisons and K-means clustering of DEGs. **a** The number of DEGs in four comparison groups, including C1 (NP0 vs. RS0), C2 (NP5 vs. RS5), C3 (NP10 vs. RS10), and C4 (NP24 vs. RS24). **b** Venn diagram of the final DEGs related to *Xoc* infection response in four comparison groups (C1 ~ C4); **c** Line charts plot of K-means clustering of DEGs and visualization of clustering heatmap

To identify genes potentially involved in *Xoc* infection and regulated by *OsROS1a*, DEGs between NP and RS lines were analyzed at four time points post-inoculation (0, 5, 10 and 24 h) through four horizontal comparisons: C1 (NP0 vs. RS0), C2 (NP5 vs. RS5), C3 (NP10 vs. RS10), and C4 (NP24 vs. RS24), respectively. C1 was used to examine the effect of RS on gene expression under normal conditions, while C2, C3, and C4 were employed to assess the impact of RS during *Xoc* infection at three post-inoculation stages. As a result, a total of 811, 337, 381, and 157 DEGs were identified in C1 (Additional file 3: Table S3), C2 (Additional file 4: Table S4), C3 (Additional file 5: Table S5), and C4 (Additional file 6: Table S6), respectively (Fig. 4a).

Notably, as *ROS1*-mediated DNA demethylation plays a crucial role in establishing genomic DNA methylation patterns and influences the methylation levels of numerous genes, the four horizontal comparisons (C1-C4) might include genes not directly related to the *Xoc* infection. Thus, we conducted six longitudinal comparisons to identify genes specifically responsive to *Xoc* infection: C5 (NP0 vs. NP5), C6 (NP0 vs. NP10), C7 (NP0 vs. NP24), C8 (RS0 vs. RS5), C9 (RS0 vs. RS10), and C10 (RS0 vs. RS24). These comparisons revealed a total of 5204 DEGs in C5 (Additional file 7: Table S7), 865 in C6 (Additional file 8: Table S8), 611 in C7 (Additional file 9: Table S9), 5572 in C8 (Additional file 10: Table S10), 2412 in C9 (Additional file 11: Table S11), and 6665 in C10 (Additional file 12: Table S12), respectively.

The final DEGs related to *Xoc* infection response between NP and RS were determined by identifying genes common to both the horizontal comparisons (C1-C4) and the longitudinal comparisons (C5-C10). Ultimately, a total of 696, 241, 273, and 108 DEGs responding to *Xoc* infection were identified in C1 (Additional file 13: Table S13), C2 (Additional file 14: Table S14), C3 (Additional file 15: Table S15), and C4 (Additional file 16: Table S16), respectively (Fig. 4b). Interestingly, more up-regulated DEGs than down-regulated genes were detected in the RS line (~ 4.5 times in C1, 3.9 times in C2, 1.9 times in C3, and 2.9 times in C4). Additionally, many genes overlapped across different stages, resulting in a total of 1080 DEGs related to *Xoc* infection between NP and RS (Fig. 4b).

### Expression patterns of DEGs related to *Xoc* infection

GO enrichment analysis of the 1080 DEGs associated with *Xoc* infection between NP and RS at four time points (Additional file 17: Table S17) revealed that these DEGs were mainly involved in 29 biological processes, 4 cellular components, and 15 molecular functions. Notably, the DEGs were significantly enriched in the response to biotic stimulus (GO:0009607), response to stress (GO:0006950) and the cell wall (GO:0005618), all of which are closely related to pathogen stress (Additional file 18: Table S18). K-means clustering analysis on all the 1080 DEGs associated with *Xoc* infection between NP and RS indicated that these DEGs were significantly grouped into 8 clusters (Fig. 4c). Each cluster exhibited a distinct gene expression pattern. Among them, cluster 3 contained the largest gene set, comprising 263 genes, followed by cluster 5 with 195 genes and cluster 2 with 165 genes. In addition, genes included in cluster 7 showed continuously upregulation following *Xoc* infection, whereas 109 genes in cluster 5 were continuously downregulated. GO analysis further revealed that these DEGs were widely involved in transcription regulation, biosynthetic process, and metabolic process (Additional file 18: Table S18). Notably, DEGs in cluster 1 were enriched in the biological processes related to defense response (GO:0006952) and response to stress (GO:0006950), as well as oxidation-reduction (GO:0055114), which are related to pathogen stress and were also enriched in clusters 2, 4, and 7. Additionally, nitrogen metabolism (GO:0051171), which is related to biochemical substance transport during the pathogen infection response, was enriched in clusters 3 and 4 (Additional file 18: Table S18).

### DNA methylation profiling of NP and RS

Genome-wide methylation profiling (BS-seq) on NP and RS line at 0, 5, 10, and 24 h post-inoculation produced more than 79 million reads in each sample with a Q20 score exceeding 96.7% (Table 2). Most reads from each sample were successfully mapped to the Nipponbare reference genome, with over 94% being uniquely mapped. Sequencing depth exceeded 31×, and more than 99.5% of cytosines were converted. The results indicated high overall sequencing quality of the eight DNA libraries.

**Table 2:** Statistics of WGBS data of different samples

Sample	Q20 Rate (%)	BS conversion ratio (%)	Total reads	Uniquely mapped reads	Mapped ratio (%)	Sequencing depth
RS-0	96.7%	99.5	85,642,302	80,284,889	93.7	32.1
RS-5	97.0%	99.7	83,345,640	79,188,903	95.0	31.7
RS-10	97.2%	99.7	86,445,980	82,447,101	95.4	33.0
RS-24	97.0%	99.6	93,883,606	89,480,695	95.3	35.8
NP-0	96.9%	99.6	79,996,554	76,391,089	95.5	30.6
NP-5	97.2%	99.7	91,195,650	87,045,059	95.5	34.8
NP-10	97.4%	99.7	90,761,908	86,074,437	94.8	34.4
NP-24	97.2%	99.7	85,376,066	80,529,292	94.3	32.21

**Fig. 5:**
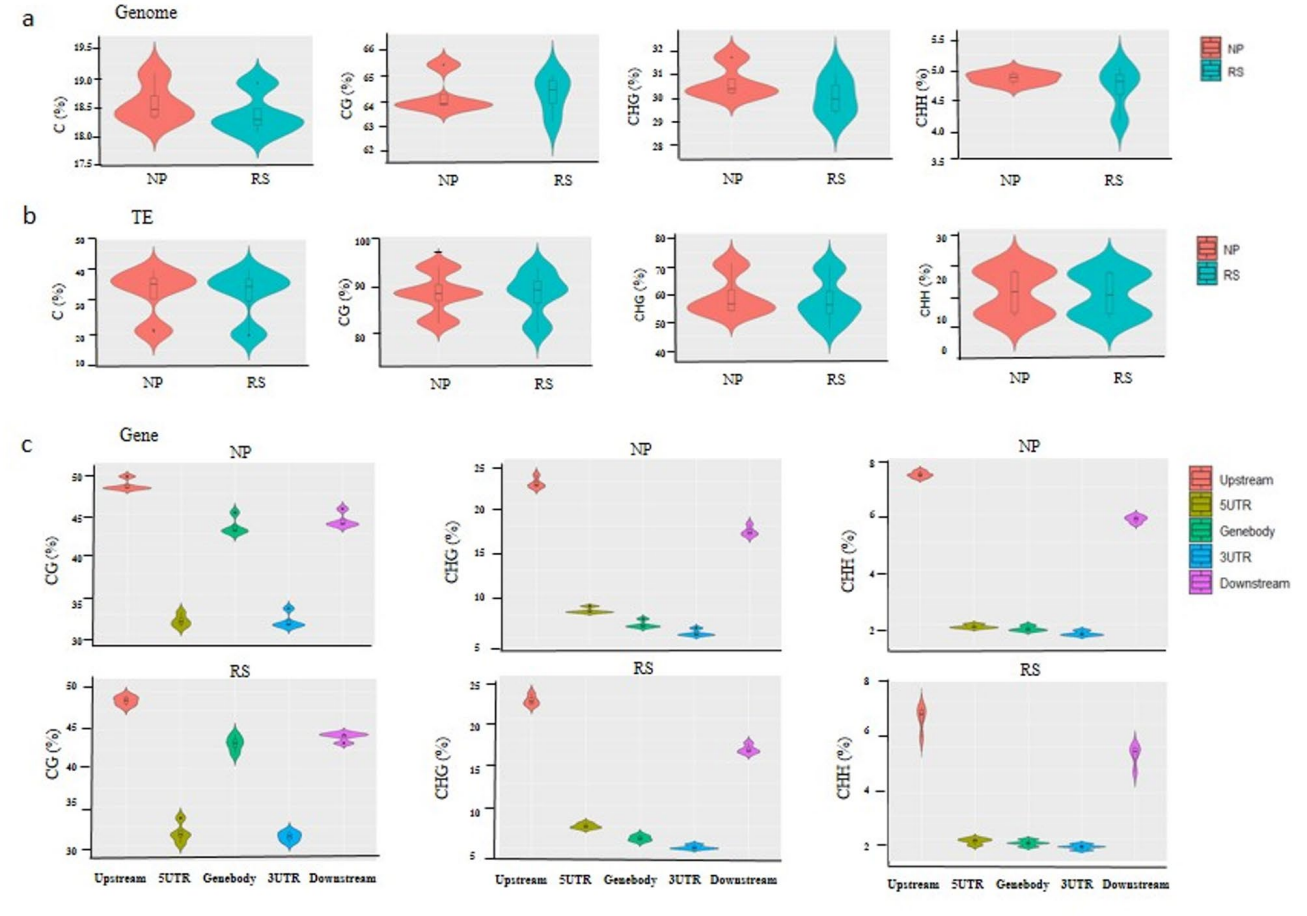
Methylation landscapes of NP and RS. **a** DNA methylation levels of wild-type (NP) and RS line in genome-wide methylated cytosine (C), and in the CG, CHG and CHH contexts; **b** The coverage of methylation level in transposon region; **c** DNA methylation patterns in different genomic regions (upstream 2 kb, 5′UTR, genebody, 3′UTR and downstream 2 kb) in the CG, CHG, and CHH contexts at NP and RS

The analysis of average methylation level revealed variations in cytosine methylation among different samples. Under *Xoc* infection, the cytosine methylation of NP and RS was similar in CG, CHG, and CHH sequence sites (where H = A, T, or C) (Fig. 5a). Notably, CG exhibited the highest methylation levels, with all samples exceeding 63%, indicating that CG methylation was the main type. When comparing the RS and NP, slightly different methylation levels were observed. In CG sites, RS showed higher methylation level compared to NP. In CHG, RS generally displayed a little lower methylation level (29.25%~30.97%) than NP (30.1%~31.71%). CHH showed relatively low overall methylation with minimal changes, slightly lower in RS compared to NP. This result indicated that suppressing the expression of *OsRos1a* altered overall methylation levels of plants, with different forms of methylation changing in response to pathogen infection. Additionally, the DNA demethylases in transposon regions were analyzed (Fig. 5b). The results showed that CG exhibited the highest methylation levels compared to CHG and CHH. When comparing the RS and NP groups, slight variations in methylation levels were observed specifically in CG. In contrast, CHG and CHH maintained similar methylation levels between NP and RS.

Further analysis of the whole-genome methylation levels within the scope of various genomic elements, including Genebody, Down2k, Up2k, 5´UTR, 3´UTR, (Fig. 5c), indicated that NP and RS had similar methylation coverage in various functional regions. Additionally, both NP and RS exhibited relatively high methylation levels in the Genebody, 3´UTR region, and Up2k region, suggesting that these areas are potentially involved in epigenetic regulation influencing gene expression.

### Comparative analysis of DEGs associated with DMGs between RS and NP during *Xoc* infection process

**Fig. 6:**
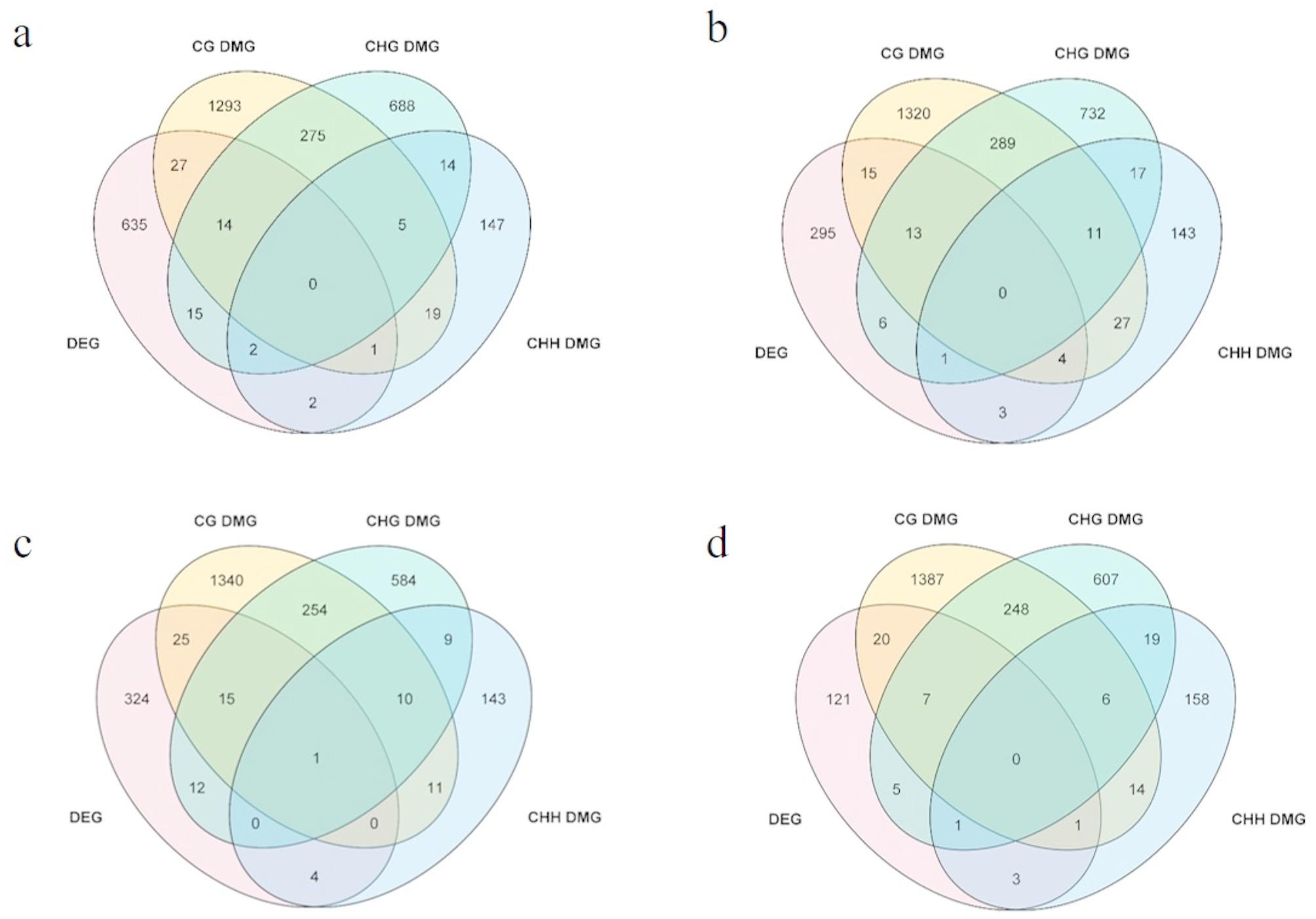
Number of DMEGs identified in different comparisons between NP and RS. **a** C1 (NP0 vs. RS0); **b** C2 (NP5 vs. RS5); **c** C3 (NP10 vs. RS10); **d** C4 (NP24 vs. RS24)

To further explore the possible association between DNA methylation and gene expression, the overlap between the DMGs and DEGs of two materials was examined at CG, CHG, and CHH sites. In comparison C1 (NP0 vs. RS0), a total of 1634, 1013 and 190 DMGs were identified under CG, CHG, and CHH contexts, respectively (Fig. 6; Additional file 19: Table S19). After merging DMEGs from CG, CHG, and CHH contexts, 61 DMEGs were obtained in C1 (Fig. 6). In C2 (NP5 vs. RS5), 1679, 1069, and 206 DMGs were determined at CG, CHG, and CHH sites, with 27 DMEGs after merging (Fig. 6; Additional file 20: Table S20). At 10 h post-inoculation, 1656, 885, and 178 DMGs were detected at CG, CHG, and CHH site in C3, resulting in 39 DMEGs after combination integration (Fig. 6; Additional file 21: Table S21). At 24 h post-inoculation, 1683, 893, and 202 DMGs were founded at CG, CHG, and CHH sites in C4, and 27 DMEGs were obtained after combination (Fig. 6; Additional file 22: Table S22). Removing overlapping DMEGs from the four comparisons yielded a total of 112 DMEGs, which were used for subsequent functional analysis (Fig. 6).

### GO annotation and KEGG pathway enrichment analysis of DMEGs

**Fig. 7:**
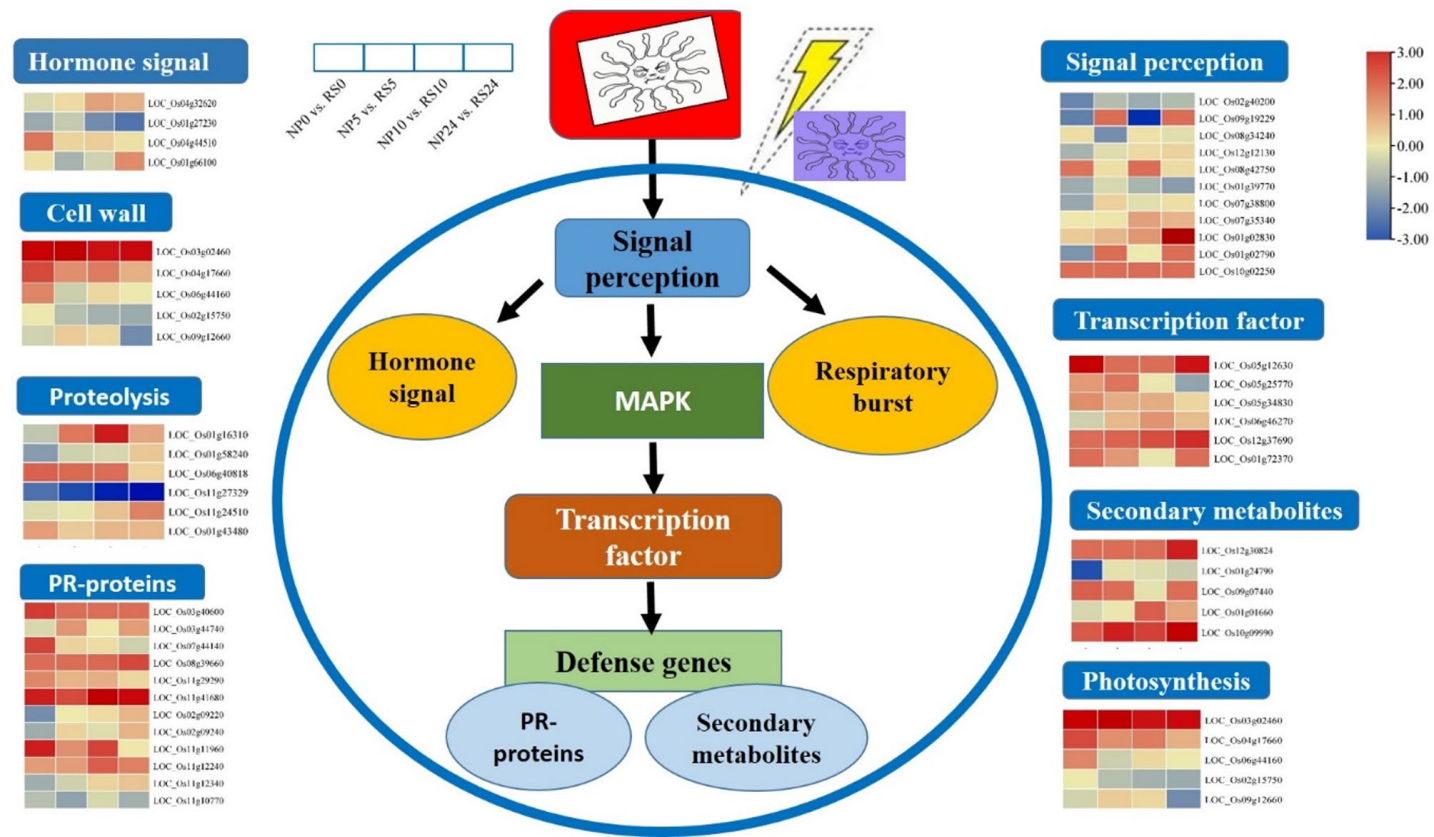
Expression changes of pathogenesis-related genes at four infection stages (0 h, 5 h, 10 h, 24 h)

**Fig. 8:**
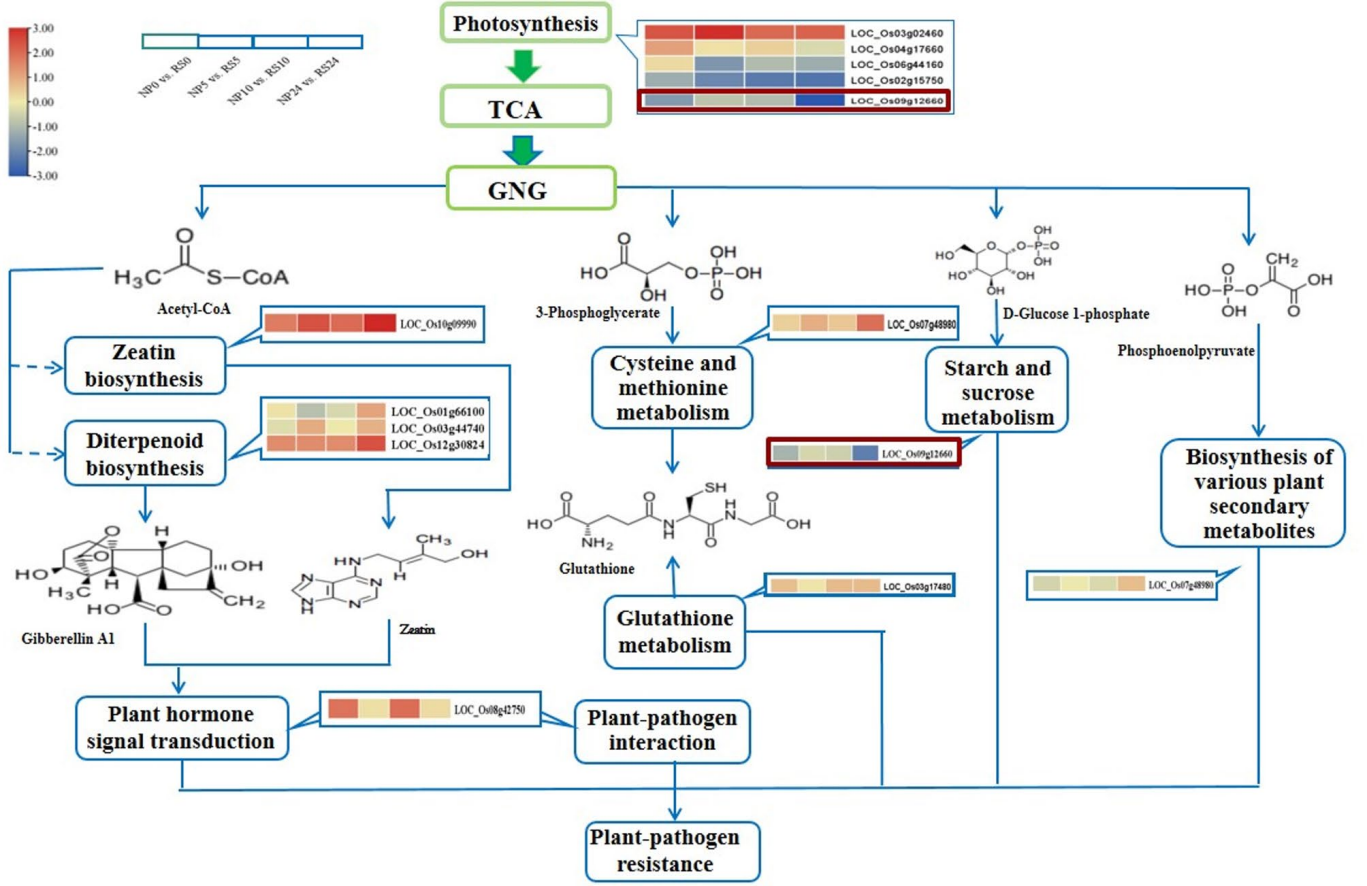
Map of KEGG pathways associated with BLS resistance. The proposed metabolic pathways were based on KEGG Pathway Database and literature. Note: The figure specifically highlights the expression levels of genes involved in several key pathways implicated in disease resistance. The red-to-blue color gradient represents the levels of gene expression, where red signifies higher expression levels and blue indicates lower expression levels. The four boxes represent four comparisons respectively (C1: NP0 vs. RS0; C2: NP5 vs. RS5; C3: NP10 vs. RS10; C4: NP24 vs. RS24)

GO analysis of the 112 overlapping DMEGs (Fig. S1; Additional file 23: Table S23) revealed that the DMEGs were enriched in 50 categories, including 21 biological processes, 15 cellular components, and 14 molecular functions. They were found to be mainly involved in biological processes, such as metabolic process (GO:0008152), cellular process (GO:0009987), response to stimulus (GO:0050896), signaling (GO:0023052), immune system process (GO:0002376). KEGG pathway enrichment analysis (Additional file 24: Table S24) indicated that the DMEGs were enriched in the pathways of purine metabolism, glutathione metabolism, plant-pathogen interaction, cysteine and methionine, alpha-linolenic acid metabolism, diterpenoid biosynthesis, photosynthesis, phenylpropanoid biosynthesis, starch and sucrose. Specifically, during *Xoc* infection, several pathogenesis-related genes exhibited significant expression changes, including hormone signal, cell wall components, degradation protein, defense gene, transcription factor, signal perception disease resistance protein, and chloroplast protein related to photosynthesis (Figs. 7 and 8). For instance, in the defense gene module, six cytochrome P450 genes (*LOC_Os03g40600*,* LOC_Os03g44740*,* LOC_Os07g44140*,* LOC_Os08g39660*,* LOC_Os11g29290*, and *LOC_Os11g41680*) were significantly up-regulated post-inoculation, whereas *LOC_Os02g09220* and *LOC_Os02g09240* were down-regulated at the 0 h stage. Additionally, various plant transcription factors played crucial roles in resistance mechanisms, such as BURP (*LOC_Os05g12630*), WRKY (*LOC_Os05g25770)*, MYB (*LOC_Os08g38800*), bHLH (*LOC_Os01g72370*), NAM (*LOC_Os05g34830*,* LOC_Os06g46270*). Notably, certain genes showed consistent up-regulation across all post-inoculation stages, including cytochrome P450 gene *LOC_Os11g41680* and transcription factor genes *LOC_Os05g12630*, *LOC_Os08g38800*, and *LOC_Os12g37690*. Additionally, the cellular component related protein thiosulfate sulfurtransferase (*LOC_Os03g02460*) and the secondary metabolite UDP-glycosyltransferase (*LOC_Os10g09990*) also showed consistent up-regulation. Conversely, *LOC_Os01g27230*, involved in hormone jasmonate synthesis-degradation, exhibited significant down-regulation at all stages post-inoculation (Fig. 7). Furthermore, most genes involved in photosynthesis were down-regulated, while genes in other gluconeogenesis metabolic pathways generally exhibited up-regulation (Fig. 8). Several chloroplast-related genes (*LOC_Os06g44160*,* LOC_Os02g15750*, and *LOC_Os09g12660*) in RS line exhibited significant down-regulation at all post-inoculation stages compared to the WT (Fig. 8). This down-regulation was consistent with the observed phenotype in the RS line, which included reduced chlorophyll content and diminished photosynthetic capacity.

## Discussion

It is widely considered that plants have developed two distinct innate immune systems: the pathogen-associated molecular pattern (PAMP)-triggered immunity (PTI) system and the effector-triggered immunity (ETI) system [43]. PTI represents a relatively weaker immune response activated by PAMPs, relying on basal defense mechanisms to limit the spread of invading pathogens [44]. ETI is triggered by polymorphic resistance proteins capable of recognizing highly variable effectors, eliciting a swift and potent response often accompanied by a hypersensitivity reaction (HR) and culminating in programmed cell death (PCD) to restrict biotrophic cellular pathogens [45]. The activation of PTI and/or ETI is finely tuned by a complex molecular and genetic network, where epigenetic regulation, including DNA methylation and demethylation, plays a critical role [2]. Photosynthesis is a primary energy source for plants and is essential for maintaining normal cellular life activities [46]. Generally, sugar signals may function through various mechanisms, potentially regulating the plant immune system and inducing systemic acquired resistance with broad-spectrum, long-lasting, and delayed effects [47–49]. In this study, we observed that the RS lines exhibited BLS symptoms more rapidly than the wild type of NP, with early signs of dark green water-stained spots on the leaves that spread along the leaf veins and gradually turn brown or yellow. Additionally, most DEGs involved in photosynthesis in RS were downregulated. Several chloroplast-related genes (*LOC_Os06g44160*,* LOC_Os02g15750*, and *LOC_Os09g12660*) in RS line exhibited significant down-regulation at all post-inoculation stages compared to the wild type of NP. This down-regulation was consistent with the observed phenotype in the RS line, which included reduced chlorophyll content and diminished photosynthetic capacity. This finding suggests that the RS lines may activate effector-triggered immunity (ETI) or pattern-triggered immunity (PTI) more rapidly and robustly than the wild type, thereby effectively restricting pathogen colonization and ultimately triggering programmed cell death (PCD) to limit the spread of biotrophic pathogens.

In the present study, we identified 112 DMEGs in response to *Xoc* using DNA methylation and transcriptome profiling. Notably, *LOC_Os09g12660* is also included in this group of DMEGs and this gene is annotated to encode the glucose-1-phosphate adenylyl transferase large subunit, a chloroplast precursor that catalyzes the synthesis of the activated glycosyl donor, ADP-glucose from Glc-1-P and ATP. Previous studies have demonstrated that ADP-glucose pyrophosphorylase (AGP) plays an essential role in starch synthesis. Among the starch biosynthetic enzymes in prokaryotes and plants, the rate-limiting enzyme, AGP catalyzes the conversion of glucose 1 - phosphate (G-1-P) and ATP into pyrophosphate (PPi) and ADP - glucose, which represents the first committed step in starch biosynthesis [50]. Recent biochemical and functional analyses have indicated that ADPase plays an active role in multiple plant biological processes, such as growth, development, environmental adaptation, and host-pathogen interactions [51]– [52]. For instance, overexpression or suppression of the small subunit of ADP-glucose pyrophosphorylase, *IbAPS*, not only affects the genes associated with chlorophyll biosynthesis, photosynthesis and carbohydrate metabolism in leaves but also influences root development and sink strength [53]. Moreover, it has been shown that infection by downy mildew (*Plasmopara viticola*) can significantly enhance AGPase activity and lead to abnormal starch accumulation in grapevine leaves [54]. Notably, *LOC_Os09g12660* was found to be a candidate gene for the QTN *qnBLS9.1*, one of nine top QTNs detected by a multi-locus GWAS analysis of 510 rice accessions tested over two seasons [33]. Additionally, the response of *LOC_Os09g12660* to *Xoc* infection was also observed in several studies [32, 55]. Taken together, these findings suggest that *LOC_Os09g12660* has strong potential to contribute to BLS resistance.

DNA methylation, a key epigenetic modification, is dynamically regulated by the opposing activities of DNA methyltransferases and DNA demethylases. This epigenetic mechanism plays a pivotal role in diverse biological processes, including gene expression regulation, genome stability maintenance, and, more recently, in the modulation of defense priming in plants. Among the DNA demethylases, *ROS1* has emerged as a critical regulator of stress tolerance and pathogen defense [13, 14, 26]. For instance, in *Arabidopsis thaliana* plants infected with beet severe curly top virus, the expression of *ROS1* is significantly upregulated, highlighting its importance in the plant’s response to viral infection [56]. *ROS1* also promotes the expression of NICOTINAMIDASE 3 (NIC3) and participates in the abscisic acid (ABA) response in *A. thaliana* plants [16]. The importance of DNA demethylation in plant immunity is further demonstrated by studies on hypo-methylated mutants, which exhibit enhanced basal resistance to (hemi) biotrophic pathogens [13, 26, 57]. For example, DNA demethylation restricts the multiplication of the bacterial pathogen *Pseudomonas syringae* pv. tomato DC3000 (Pst) in *Arabidopsis* leaves. In *ros1* mutants, a slight increase in bacterial growth was observed and this increase is associated with the activation of a salicylic acid-dependent defense response, highlighting the role of *ROS1*-dependent DNA demethylation in antibacterial resistance [13]. Additionally, recent studies found that the Pol V mutant (*nrpe1*) and the *rdd* triple demethylase mutant (*ros1 dml2 dml3*) increased the susceptibility to *Fusarium oxysporum* due to a lack of *RdDM*-induced DNA demethylation at relevant defense genes [58]. Interestingly, *nrpe1* and *ros1* mutants exhibit opposite resistance phenotypes to *Hyaloperonospora arabidopsidis* and *Plectosphaerella cucumerina* [25]. The hypo-methylated *nrpe1* mutant, which is impaired in RNA-directed DNA methylation, demonstrates increased susceptibility to the necrotrophic pathogen *Plectosphaerella cucumerina*. This susceptibility is associated with reduced sensitivity to jasmonic acid (JA)-inducible gene expression. Conversely, the hyper-methylated *ros1* mutant, which is deficient in DNA demethylation, shows enhanced resistance to necrotrophic pathogens. This increased resistance is not linked to greater responsiveness to JA-inducible gene expression. Therefore, basal resistance against *Hyaloperonospora arabidopsidis* and *Plectosphaerella cucumerina* is not regulated by RdDM-induced ROS1 activity. Instead, it is controlled by the opposing effects of RdDM- and ROS1-dependent DNA demethylation on relevant defense genes [25].

**Fig. 9:**
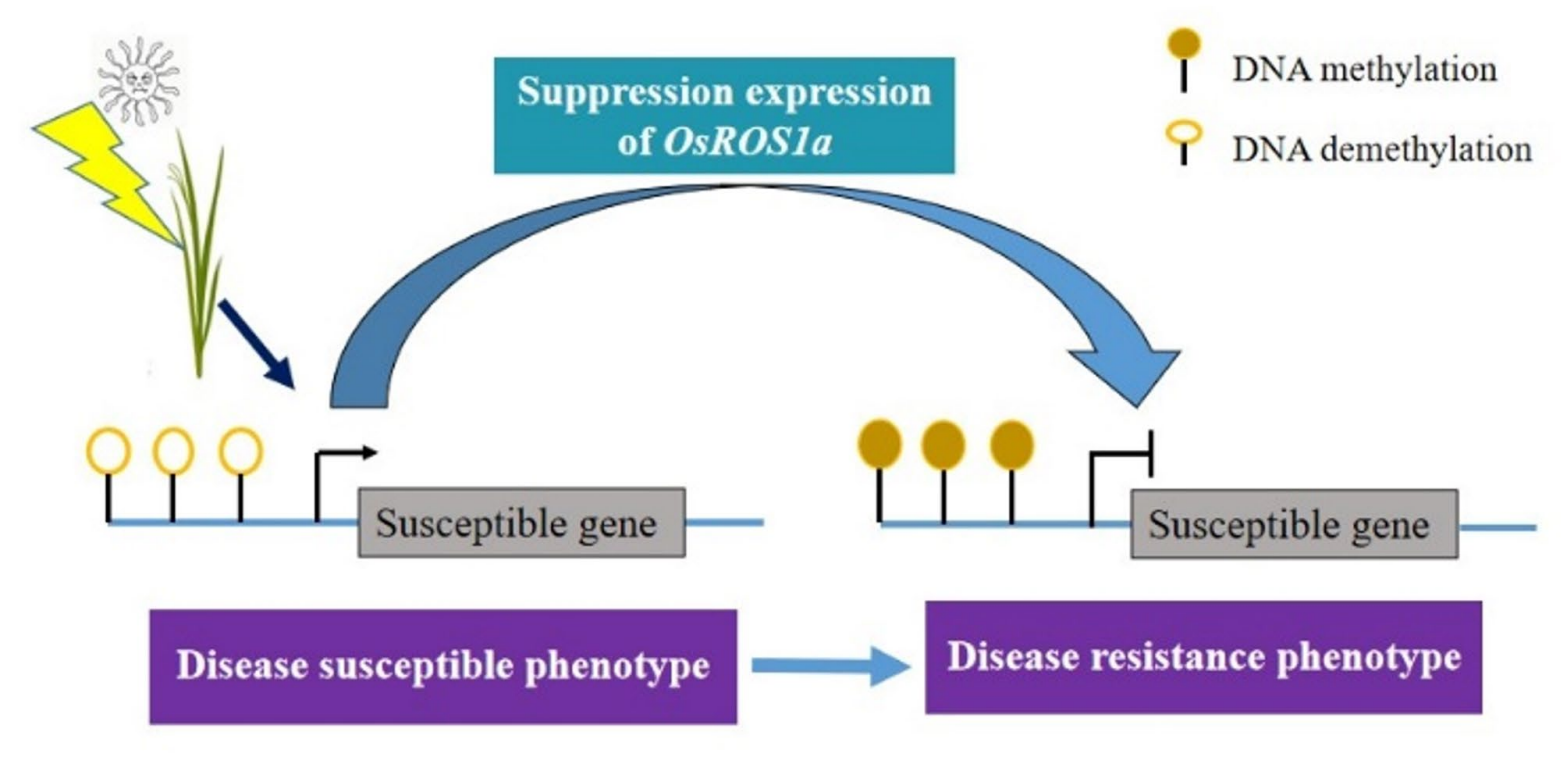
Hypothetical model of BLS resistance mechanism regulated by *OsROS1a*. It is speculated that certain susceptibility genes associated with BLS disease are suppressed through hyper-methylation, a process triggered by the suppression of *OsROS1a* expression by RNAi. This epigenetic modification results in an enhanced resistance to BLS in the RS line

In our study, the RS lines exhibited increasing resistance to BLS caused by *Xoc* infection, suggesting that DNA demethylation plays a significant role in modulating the defense-related transcriptome through trans-regulatory mechanisms. Previous study has demonstrated that BLS is caused by TALE-carrying *X. oryzae* pv. oryzicola [59]. Furthermore, the direct interaction of several TALEs with the transcription factor *TFIIAg5* is essential for the activation of disease susceptibility genes [59, 60]. Given this, we hypothesize that the BLS resistance mechanism regulated by *OsROS1a* involves the suppression of specific susceptibility genes in the RS line. These genes are likely hyper-methylated due to the RNAi-mediated suppression of *OsROS1a* expression, which prevents their activation by TALEs (Fig. 9). The relationship between specific DNA methylation patterns and gene expression is complex, and to systematically unravel and dissect the underlying mechanisms, further studies will be necessary.

This study explores the resistance mechanism of BLS from the perspective of methylation. By integrating both methylome and transcriptome analyses, it illuminates the epigenetic regulation of *OsROS1a* in rice’s resistance to BLS. This research offers a novel perspective for the study of BLS resistance. The findings of this study provide a foundation for developing rice varieties with enhanced resistance to this disease. Genome editing tools offer precise and efficient methods to enhance BLS resistance by targeting *OsROS1a* or its downstream genes. For instance, knocking down *OsROS1a* or editing its regulatory regions could change DNA methylation levels at specific loci, thereby activating defense-related genes or suppressing susceptibility genes, and conferring resistance. Alternatively, directly editing downstream targets, such as *LOC_Os09g12660*, could enhance resistance without altering the broader epigenetic landscape.

## Conclusion

In conclusion, this study demonstrates the critical role of *OsROS1a* in modulating rice resistance to bacterial leaf streak (BLS) caused by *Xanthomonas oryzae* pv. oryzicola (*Xoc*). The RNA interference-mediated knockdown of *OsROS1a* in the Nipponbare rice variety resulted in significantly reduced BLS lesion lengths, confirming the gene’s significance in defense against this pathogen. Through whole-genome bisulfite sequencing (WGBS) and RNA sequencing (RNA-seq), we identified significant changes in both DNA methylation and gene expression. Specifically, 112 differentially methylated and expressed genes (DMEGs) were found to overlap between the methylome and transcriptome datasets. These DMEGs were associated with crucial biological pathways including biotic stress responses, hormone signaling, and secondary metabolite synthesis. KEGG pathway enrichment analysis further revealed their involvement in key processes such as plant-pathogen interactions and metabolic pathways. Notably, the *LOC_Os09g12660* gene, which encodes a chloroplast precursor involved in glycosyl donor synthesis, emerged as a potential target for improving BLS resistance. These findings enhance our understanding of the molecular mechanisms by which DNA methylation influences BLS resistance in rice.

## Supplementary Information

Below is the link to the electronic supplementary material.

Supplementary Material 1

Supplementary Material 2

Supplementary Material 3

Supplementary Material 4

Supplementary Material 5

Supplementary Material 6

Supplementary Material 7

Supplementary Material 8

Supplementary Material 9

Supplementary Material 10

Supplementary Material 11

Supplementary Material 12

Supplementary Material 13

Supplementary Material 14

Supplementary Material 15

Supplementary Material 16

Supplementary Material 17

Supplementary Material 18

Supplementary Material 19

Supplementary Material 20

Supplementary Material 21

Supplementary Material 22

Supplementary Material 23

Supplementary Material 24

Supplementary material 25

## Data Availability

Sequence data that support the findings of this study have been deposited in the NCBI Sequence Read Archive repository under the accession number PRJNA1149540.

## Abbreviations

BLS: Bacterial leaf streak
Xoc: *Xanthomonas oryzae* pv. Oryzicola
FPKM: Fragments per Kilobase of transcript sequence per Millions base pairs sequenced
RS: OsROS1a-RNAi
NP: Nipponbare plants
WGBS: Whole-genome bisulfite sequencing
RNA-seq: RNA-sequencing
GO: Gene ontology
RdDM: RNA-directed DNA methylation
Pst: *Pseudomonas syringae* pv. Tomato
DEGs: Differentially expressed genes
DMRs: Differentially methylated regions
DMGs: Differentially methylated genes
DMEGs: Differentially methylated and expressed genes
qRT-PCR: Quantitative real-time PCR
