# Supplementary material for: Exploring the molecular mechanism of OsROS1a in regulating resistance to bacterial leaf streak through transcriptome and DNA methylation profiling in rice (Oryza sativa L.)
Source: BMC Genomics. 2025 Aug 1;26:713. doi: 10.1186/s12864-025-11895-1 (PMC12315458; doi:10.1186/s12864-025-11895-1)
Supplement: Supplementary file 1 — Supplementary Material 1 [file 12864_2025_11895_MOESM1_ESM.docx]

Table S1 Primers used in vector construction and transgenic plant identification

| Application | Primer name | Primer Sequence (5′-3′)^a^ |
| --- | --- | --- |
| RNAi template | Rosi-F | cAGTGGTCTCacaacatgcaggattttggacaatggctgc |
|  | Rosi-R | cAGTGGTCTCacaggtgacttcagctcagatctgcagtgt |
|  | loop -F | cgatGGTCTCacctgcaggtctagtttttctccttc |
|  | loop -R | cAGTcgatGGTCTCagcccgggctctgtaactatcatcat |
| Hygromycin | RosiC-F  RosiC-R  Hyg-R | cAGTGGTCTCagggctgacttcagctcagatctgcagtgt  cAGTGGTCTCatacaatgcaggattttggacaatggctgc  ACGGTGTCGTCCATCACAGTTTGCC |
|  | Hyg-F | TTCCGGAAGTGCTTGACATTGGGGA |

^a^ Restriction site sequences are underlined
