# Supplementary material for: Exploring the molecular mechanism of OsROS1a in regulating resistance to bacterial leaf streak through transcriptome and DNA methylation profiling in rice (Oryza sativa L.)
Source: BMC Genomics. 2025 Aug 1;26:713. doi: 10.1186/s12864-025-11895-1 (PMC12315458; doi:10.1186/s12864-025-11895-1)
Supplement: Supplementary file 2 — Supplementary Material 2 [file 12864_2025_11895_MOESM2_ESM.docx]

Table S2 Primer sequences for qRT-PCR

| Primer Name | Forward（5’-3’） | Reverse（5’-3’） |
| --- | --- | --- |
| LOC_Os05g12400 | CGCTGCTGTCCTACTCCTTT | CCTTCTCGAGGAAGAACACG |
| LOC_Os11g11890 | TCACAGCTCACAAGCAAACC | GTGCTGGATTCACCCTTCAT |
| LOC_Os02g34760 | GAGCTTTGCGTTTTTCCTTG | CTTCTCTCTCGCCTTGCATT |
| LOC_Os07g01820 | CACAAACACCTCATGGGAGA | TCCTGCAGTGACCTCTCCTT |
| LOC_Os11g27329 | CTGGCCTAACCCACGAGATA | TCTTGGAGCCTTCCTGTGTT |
| LOC_Os01g11900 | CCAAGGGCGTGTTGATTCAT | AGCCATAAATGCAGAGCTGG |
| Actin | gattgccaaggctgagtacga | aaaagaagaaacaagcaggagga |
