# Supplementary figures and images for: Exploring the molecular mechanism of OsROS1a in regulating resistance to bacterial leaf streak through transcriptome and DNA methylation profiling in rice (Oryza sativa L.)
Source: BMC Genomics. 2025 Aug 1;26:713. doi: 10.1186/s12864-025-11895-1 (PMC12315458; doi:10.1186/s12864-025-11895-1)

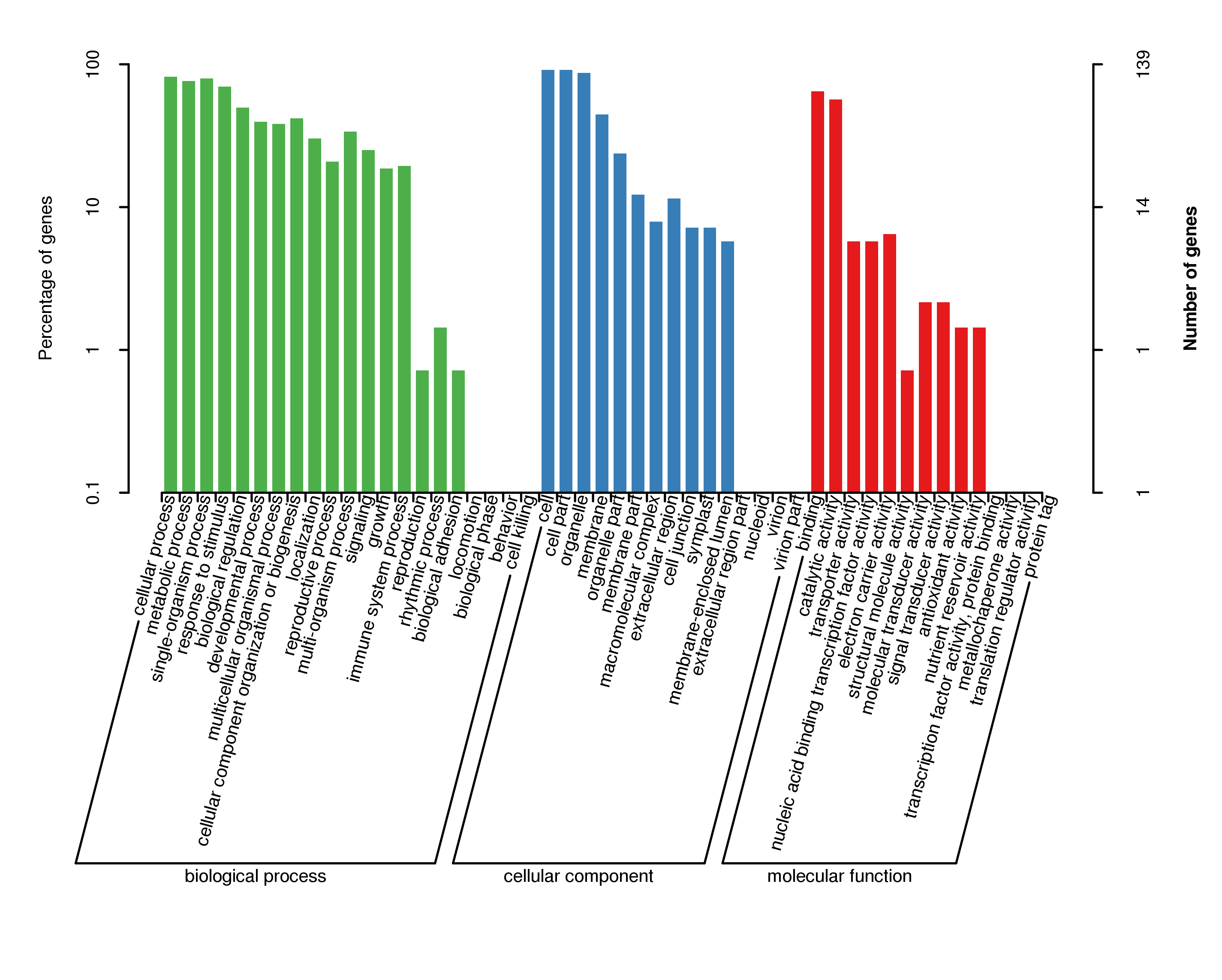

Supplement: Supplementary file 25 — Supplementary material 25 [file 12864_2025_11895_MOESM25_ESM.png]
